# Highly Efficient Synthesis of Type B Gelatin and Low Molecular Weight Chitosan Nanoparticles: Potential Applications as Bioactive Molecule Carriers and Cell-Penetrating Agents

**DOI:** 10.3390/polym13234078

**Published:** 2021-11-24

**Authors:** Cristina Gonzalez-Melo, Andres J. Garcia-Brand, Valentina Quezada, Luis H. Reyes, Carolina Muñoz-Camargo, Juan C. Cruz

**Affiliations:** 1 Department of Biomedical Engineering, Universidad de Los Andes, Bogotá 111711, Colombia; c.gonzalez19@uniandes.edu.co (C.G.-M.); aj.garcia14@uniandes.edu.co (A.J.G.-B.); v.quezada@uniandes.edu.co (V.Q.); 2 Product and Process Design Group (GDPP), Department of Chemical and Food Engineering, Universidad de los Andes, Bogotá 111711, Colombia

**Keywords:** LMW chitosan, type B gelatin, nanoparticles, synthesis

## Abstract

Gelatin and chitosan nanoparticles have been widely used in pharmaceutical, biomedical, and nanofood applications due to their high biocompatibility and biodegradability. This study proposed a highly efficient synthesis method for type B gelatin and low-molecular-weight (LMW) chitosan nanoparticles. Gelatin nanoparticles (GNPs) were synthesized by the double desolvation method and the chitosan nanoparticles (CNPs) by the ionic gelation method. The sizes of the obtained CNPs and GNPs (373 ± 71 nm and 244 ± 67 nm, respectively) and zeta potential (+36.60 ± 3.25 mV and −13.42 ± 1.16 mV, respectively) were determined via dynamic light scattering. Morphology and size were verified utilizing SEM and TEM images. Finally, their biocompatibility was tested to assure their potential applicability as bioactive molecule carriers and cell-penetrating agents.

## 1. Introduction

Nanoparticles (NPs) have been used in many applications based on their small size, surface area, and morphology [1,2,3]. NPs exhibit special attention for their application in diagnosis, imaging, drug delivery, and bioactive compound encapsulation [4]. In this regard, NPs have been demonstrated to improve drug–target approaches and compound delivery, improving dissolution kinetics and controlled release [3]. Furthermore, the chemically reactive surface functional groups have allowed for functionalization with a wide range of chemical and biochemical molecules such as peptides, nucleic acids, and small drugs that makes them suitable to overcome physiological barriers such as the intestinal lumen (pH = 5.5–7.0) and the blood–brain barrier [3,5,6,7].

Polymeric nanoparticles (PNPs) have attracted significant attention given their high biocompatibility and degradation times, making them optimal as adjuvants in drug and bioactive compound carriers, gene-editing, and even cell biochemical pathways detection [8,9]. PNPs have been synthesized from both synthetic and natural polymers to yield a tunable spectrum of properties such as surface charge, shape, reactive functional groups, and size [10].

PNPs provide a cost-effective way for the encapsulation of bioactive compounds for oral delivery (e.g., micronutrients, enzymes, and antioxidants). In this regard, these encapsulation techniques have demonstrated promising results to overcome extreme physiological conditions commonly found along the gastrointestinal tract (GIT) that usually involve the exposure to oxygen, changes in pH (usually from 1.3 to 7.4) [11], shear stress, and the presence of degrading enzymes [12]. In fact, the encapsulation or immobilization of bioactive compounds provide absorption-enhancing properties for functional food manufacturing while maintaining their structural stability, especially as they pass through the GIT [13]. Transit through the GIT negatively impacts the stability of bioactive compounds, which results in poor bioavailability and low permeability across the intestinal barrier [12,14].

Among the synthetic polymers, poly(ethylene glycol) and poly(lactic–co-glycolide) have been tested to deliver drugs, nutraceuticals, and bioactive molecules and macromolecules [15]. However, they have demonstrated better performance in vitro and in vivo when tissue-resemble materials are employed for their synthesis [16]. For this reason, this approach has been widely used for applications that require high biodegradability, biocompatibility, non-antigenicity, low cost, and even availability from renewable sources, as is the case of chitosan and gelatin [16].

Gelatin nanoparticles (GNPs) have received particular attention due to their collagen precedence, which confers an amino acidic-based structure comparable to proteins and peptides [16,17]. Additionally, the remarkable properties of chitosan nanoparticles (CNPs) have been attributed to their polysaccharide structure that confers antimicrobial [18,19,20], mucoadhesive [21], and analgesic properties [22].

Synthesis of PNPs of natural origin is generally centered on polymerization or depolymerization by desolvation, emulsion-based synthesis, ionic gelation, and nanoprecipitation [23,24]. However, the complex downstream processing for purification represents one of their main issues, limiting their scalability and industrialization. This is mainly due to their low density and small size, limiting conventional unit operations such as precipitation, centrifugation, and filtering. This ultimately leads to significantly low synthesis yields [25].

Conversely, this work offers an alternative synthesis method of higher yield and low-cost for type B gelatin and low molecular weight (LMW) chitosan NPs. We introduced two-step desolvation and ionic gelation synthesis methods with enhanced recovery and purification steps. The obtained NPs were characterized in terms of shape, morphology, and surface charge. Moreover, we conducted preliminary biocompatibility assays to investigate their potential as bioactive molecule carriers and cell-penetrating agents.

## 2. Materials and Methods

### 2.1. Materials

Hydrochloric acid (HCl, 37%, CAS 7647-01-0), glutaraldehyde (GTA, 25%, CAS 111-30-8), acetone (99.5%, CAS 67-64-1), glacial acetic acid (99.7%, CAS 64-19-7), and sodium hydroxide (NaOH, 98%, CAS 1310-73-2) were purchased from PanReac AppliChem (Chicago, IL, USA). Low molecular weight (LMW) chitosan (50–190kDa, deacetylation degree of 75–85%, CAS 9012-76-4), phosphate buffer saline (PBS-1X), thiazolyl blue tetrazolium bromide (MTT, CAS 57360-69-7), dimethyl sulfoxide (DMSO, 99%, CAS 67-68-5), and Dulbecco’s modified Eagle’s medium (DMEM) were purchased from Sigma-Aldrich (St. Louis, MO, USA) and Type B gelatin from the local store Químicos Campota (Bogotá, Colombia). Fetal bovine serum (FBS) was obtained from Biowest (Riverside, MO, USA). Trypsin EDTA was obtained from Lonza (Riverside, MO, USA).

### 2.2. Synthesis of Type B Gelatin and LMW Chitosan Nanoparticles

**Figure 1:**
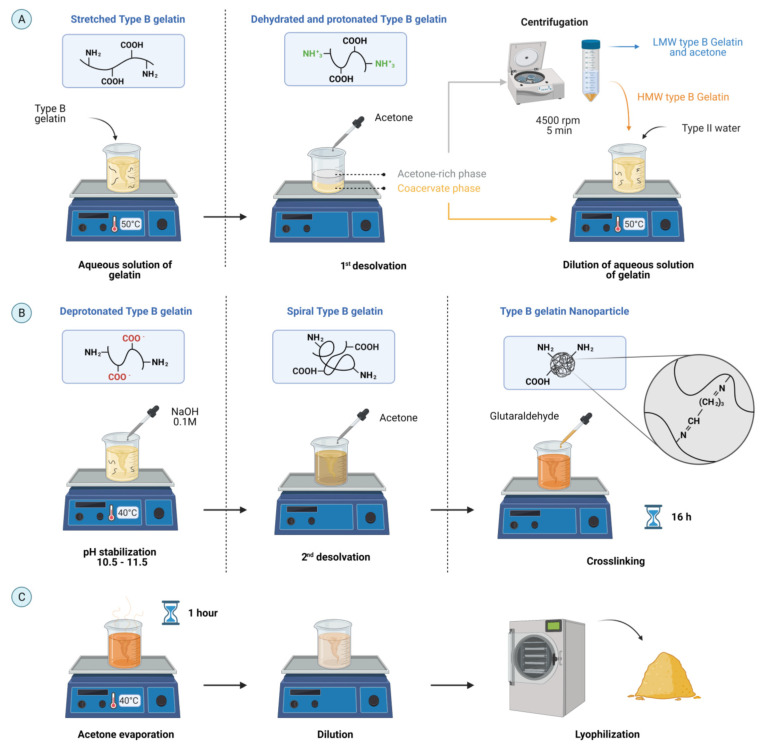
Schematic for the synthesis of type B gelatin NPs (GNPs) by a two-step desolvation method with acetone. (**A**) Type B gelatin was dissolved, dehydrated, and protonated by a first desolvation step. (**B**) Then, HMW type B gelatin was deprotonated to then induce a controlled gelatin coiling and crosslinking. (**C**) GNPs recovery (created with BioRender.com, accessed on 12 October 2021).

A two-step desolvation method was used to synthesize GNPs (Figure 1) [26]. Type B gelatin (5% p/v) was dissolved in type II water at 50 °C by magnetic stirring. Then, acetone at a volume ratio of 1:1 was dripped to the mixture and left still for 5 min to dehydrate gelatin and induce coiling by the prevalence of positive charges in protonated amine (–NH2) functional groups [27,28]. The supernatant, rich in acetone, was collected and centrifuged for 3 min at 4500 RPM to recover the remaining high molecular weight (HMW) gelatin. Then, the remaining pellet was diluted in type II water (volume ratio of 1:1 with acetone) and reincorporated to the coacervate phase by mixing at 50 °C for 20 min. The pH was adjusted between 10.5–11.5 with 0.1 M NaOH to ionize the pendant carboxyl (–COOH) functional groups of acidic amino acids (i.e., glutamic and aspartic acids) [28].

Then, the gelatin was desolvated again by incorporating acetone (6 mL of acetone per milliliter of the initial gelatin solution) at 2 mL/min at room temperature to change their conformation from stretched to spiral by a controlled restoration of charges and to prevent precipitation [27,29]. Subsequently, chemical crosslinking of the –NH2 of gelatin was induced by adding 4 µL of GTA per milliliter of the starting solution and allowing it to react for 16 h. Excess acetone was evaporated at 40 °C for 1 h under continuous magnetic stirring followed by the dilution of the mixture (1:3 *v/v*) with type II water to avoid agglomeration. Finally, the GNPs were recovered by lyophilization and stored at 4 °C until further use.

**Figure 2:**
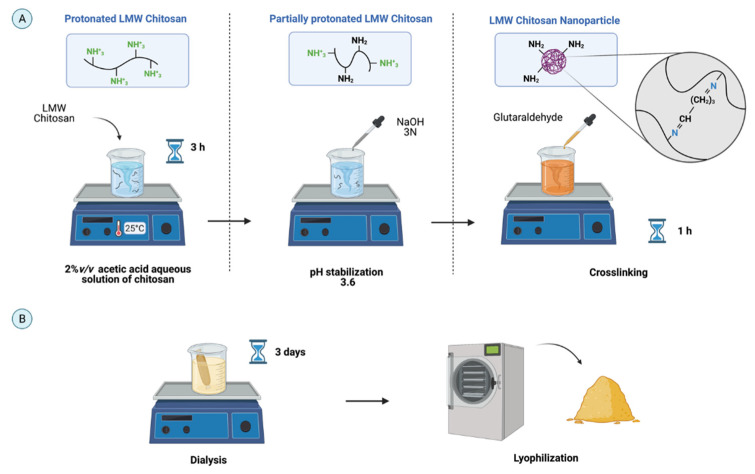
Schematic for synthesizing low molecular weight (LMW) chitosan NPs (CNPs) by the ionic gelation method in aqueous 2% *v/v* acetic acid. (**A**). LMW chitosan was protonated to achieve dissolution and deprotonated to control aggregation before chemical crosslinking with GTA to form the NPs. (**B**). CNP purification and recovery by three-day dialysis and lyophilization (created with BioRender.com, accessed on 12 October 2021).

The ionic gelation method was used as a reference to design the new synthesis protocol of CNPs [30]. Chitosan (2.4 mg/mL) was dissolved in acetic acid (2% *v/v*) by continuous magnetic stirring for 3 h to protonate the pendant –NH2 groups of monomers and thus increase its solubility (Figure 2). The pH was adjusted to 3.6 with NaOH 3 N to induce partial charge restoration. To obtain the CNPs, chitosan chains were crosslinked with 1.2 μL of GTA per millimeter and stirred for 1 h. To recover the CNPs and remove excess solvents, the mixture was dialyzed against Type II water using a 2 kDa membrane (Sigma-Aldrich, St. Louis, MO, USA) for three days at room temperature, lyophilized, and finally stored at 4 °C. For both syntheses, experiments were conducted in triplicate to calculate the synthesis yield from the initial weight of the polymer and the weight of lyophilized NPs.

### 2.3. Characterization

The size distribution and surface charge in aqueous solution were measured with the aid of a dynamic light scattering (DLS) instrument Zeta-sizer Nano (Malvern Panalytical, Malvern, UK). Microscopic inspection of the nanosized structure, morphology, and shape was achieved via transition electron microscopy (TEM) in a Tecnai F30 (FEI Company, Fremont, CA, USA) and scanning electron microscopy (SEM) in a JSM6490-LV TESCAN (JEOL, Tokyo, Japan) at ×3000 and ×400 magnifications with a 10 kV accelerating voltage. Moreover, NP morphologies in solution were characterized by cryogenic-SEM by dripping 5 µL of NPs suspensions into liquid nitrogen.

### 2.4. Biocompatibility

Biocompatibility characterization following the international standard ISO10993 was included to ensure their safe use for potential biomedical applications such as therapeutic biomolecule carriers [31]. The hemolytic activity was tested by measuring erythrocyte lysis after isolation from a healthy human donor (4.2 × 10^6^ erythrocytes) using EDTA collection tubes. The samples were obtained with the approval of the Ethical Committee at the Universidad de Los Andes (minute number 928-2018, which also contained the informed consent signed by subjects). Separation of plasma and white blood cells was achieved by removing the supernatant after centrifugation at 1800 RPM for 5 min at room temperature. The low-density phase containing the erythrocytes was resuspended and washed three times with NaCl (0.9% *w/v*) and once with PBS-1X. A diluted erythrocyte stock solution was prepared by adding 1 mL of the isolated erythrocytes in 9 mL of PBS-1X. Then, serial dilutions of 1:2 of GNPs and CNPs from 200 to 12.5 µg/mL were prepared in PBS-1X and exposed to the same volume of the erythrocyte stock in a 96-well microplate. The contact was allowed for 1 h at 37 °C, and the samples were then centrifugated at 1800 RPM for 5 min. Finally, 100 µL of each supernatant was transferred to a new microplate and read at 450 nm in a microplate spectrophotometer (Thermo Scientific™, Waltham, MA, USA). PBS-1X and Triton X-100 were used as positive and negative controls, respectively. The experiments were conducted in triplicate.

To avoid coagulation, the platelet aggregation induced by the GNPs and CNPs was tested on isolated platelets from freshly drawn blood from a healthy human donor in 3.2% citrate blood tubes. Then, blood was centrifugated to obtain the platelet-rich plasma (PRP) at 1000 RPM for 15 min at room temperature. Samples of serial dilutions of GNPs and CNPs from 200 to 12.5 µg/mL were prepared in PBS before exposure to PRP (1:1 *v/v*) for a final volume of 100 µL. Thrombin (Thb) and PBS-1X were used as the positive and negative controls, respectively. The treatments were incubated for 5 min at room temperature, and the absorbance was recorded at 620 nm in a microplate spectrophotometer.

Cytocompatibility was confirmed by measuring the metabolic activity of Vero cells (ATCC^®^ CCL-81) by quantifying the conversion of 3-[4,5-dimethylthiazol-2-yl]-2,5-diphenyltetrazolium bromide (MTT) to formazan in the mitochondria. Briefly, 100 µL of a cell stock of 100,000 cells/mL in DMEM media supplemented with 10% FBS were deposited in a 96-well microplate for a cell density of 10,000 cells/well and incubated at 37 °C and 5% CO_2_ for 24 h. After incubation, the media was removed, and 100 µL of serial dilutions 1:2 (i.e., 200–12.5 mg/mL) of concentrated stocks of GNPs and CNPs in DMEM media were exposed and incubated 37 °C and 5% CO_2_ for 24 h and 48 h. Then, MTT reagent was added and allowed to react for 2 h before replacing the media with 100 µL of DMSO to dissolve the formazan crystals. Finally, absorbance was measured at 595 nm in a microplate spectrophotometer to calculate cell viability.

## 3. Results and Discussion

### 3.1. Synthesis Yield

For both CNPs (92.60% ± 0.66%) and GNPs (89.95% ± 1.13%), the obtained synthesis yield was about 6% higher than the synthesis methods reported previously (Table 1). Although the nanoprecipitation method reports a yield of up to 95%, the resultant GNPs exhibited a larger size than the two-step desolvation method [32,33] and required an excipient stabilizer to avoid aggregation [34,35].

The high synthesis yield obtained here can be attributed to the purification and recovery alternatives based on solvent dialysis and lyophilization. This, considering that liquid-phase surface tension hinders precipitation of nanosized structures with low-density, led to the recovery of only the larger nanoparticles. In contrast, the smaller ones are commonly discarded [36]. Additionally, dialysis and lyophilization are optimal alternatives for small-size nanoparticle recovery without compromising their final physicochemical properties, as discussed below.

**Table 1:** Comparison of PNP yield synthesis.

Type	Method	Nanoparticle Yield (%)	References
	Two-step desolvation	89.94% ± 1.13%	This study
GNPs	One-step desolvation	≤83%	[37,38]
	Two-step desolvation	1.5–83%	[38,39,40,41,42]
	Nanoprecipitation	20–95%	[43,44,45,46,47]
	Ionic gelation	92.60% ± 0.66%	This study
CNPs	Ionic gelation	≤86%	[48,49,50,51]
	Emulsion cross-linking	32–51%	[52]
	Spray drying	13–85%	[53,54,55]

### 3.2. Characterization of Nanoparticles

**Figure 3:**
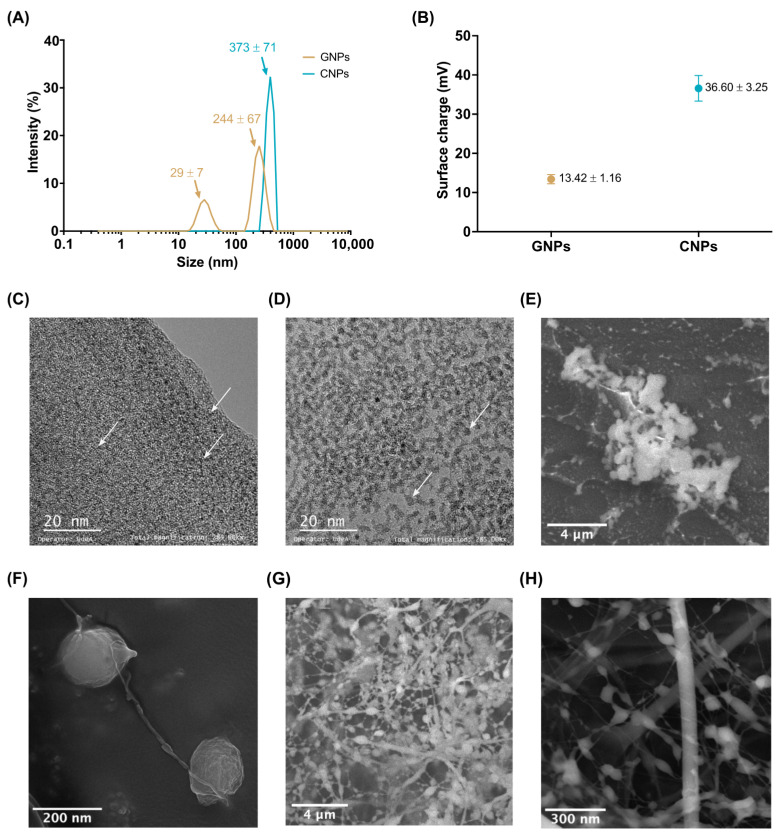
Characterization of GNPs and CNPs. (**A**) Hydrodynamic average diameter. CNPs were close to 373 nm, and GNPs to 244 nm and 29 nm. (**B**) Zeta-potential (absolute value) of GNPs (−13 mV) and CNPs (+37 mV). TEM image of GNPs (**C**) showed a nominal size of 2 nm while CNPs (**D**) of 5 nm. (**E**) SEM image of GNPs. White dots confirm NP size below 100 nm and up to 400 nm agglomerates with a spherical shape. (**F**) SEM image of CNPs. The visualized sample shows 170 nm NPs with an elongated structure. Cryogenic-SEM of an aqueous solution of GNPs (**G**) and CNPs (**H**). Round-shaped morphology, nanofibers, and agglomerate formations. Aggregates of NPs and round-shaped morphology were observed together with fiber-like structures interconnected due to absorbed water between NPs. White arrows point to the individual polymeric NPs.

GNPs showed two average hydrodynamic diameters of 244 ± 67 nm and 29 ± 7 nm, while CNPs showed a single mean hydrodynamic diameter of 373 ± 71 nm in an aqueous medium (Figure 3A). TEM images show an average nominal size of 2 nm for GNPs (Figure 3C) and 5 nm for CNPs (Figure 3D). GNP and CNP morphologies observed by SEM were spherical (Figure 3C,D) and exhibited an average size consistent with that obtained by DLS (Figure 3E,F).

Cryogenic-SEM (Figure 3G,H) of the NPs in aqueous media showed an interconnected network that could be attributed to the interaction of polymeric chains as a result of retained water between adjacent NPs [56]. In the case of GNPs, this behavior is most likely due to the effect of RGD (arginyl-glycyl-aspartic acid) regions present in the main backbone of, which may cause aggregation and self-assembly [56]. However, CNPs tend to form aggregates due to the ionic strength of the glucosamine units [57].

Zeta-potentials of the GNPs (−13.42 ± 1.16 mV) and CNPs (+36.60 ± 3.25 mV) in type II water at neutral pH indicate superior colloidal stability (Figure 3B). In both cases, the average zeta-potential is related to ionizable functional groups in the monomers of the polymeric chains after their extraction processes. In this regard, the negative zeta-potential of the GNPs is explained by the alkali extraction of type B gelatin from collagen that induces an isoelectric point between 4.8–5.4 due to partial deamination of asparagine and glutamine [58,59]. This suggests that if GNPs are used as cell-penetrating agents, they are more likely to bind to cationic sites in the cell membrane via electrostatic interactions. This phenomenon generates a localized neutralization that favors the adsorption and clustering of other free NPs that bend the membrane and ultimately internalize through endocytosis, facilitating the delivery of intracellular therapeutic molecules, especially through absorptive epithelial cells (enterocytes) found along the intestinal lumen [60,61].

The deacetylation of chitin explains the zeta-potential of CNPs during their extraction process [62], where the resulting polysaccharide contains a large number of pendant amine groups that might protonate and confer a positive surface charge with high colloidal stability in aqueous solvents [62,63]. Additionally, if CNPs are used for cell penetration, they are expected to interact with negatively charged sulfated proteoglycans that can be found ionized on cell membranes to facilitate internalization [60].

The measured zeta-potentials of the GNPs and CNPs is consistent with previously reported studies [29,64,65,66]. In particular, CNP zeta-potential was reported between +35 ± 6.53 and +47 ± 4.37 mV [67], while the zeta-potential of GNPs in a pH between 6 and 8 is close to −13 mV [68]. Moreover, the two types of NPs produced here appear suitable as biomolecule carriers for oral delivery as evidenced by their possible destabilization as they reach the intestinal lumen, thereby facilitating the targeted release of cargoes [61].

### 3.3. Biocompatibility

To evaluate the potential of NPs as cell-penetrating and bioactive-carrier nanoplatforms, in vitro biocompatibility was tested following the international standard ISO10993. Accordingly, platelet aggregation, hemolysis, and cytotoxicity in epithelial-like cells (i.e., Vero cells) were evaluated.

**Figure 4:**
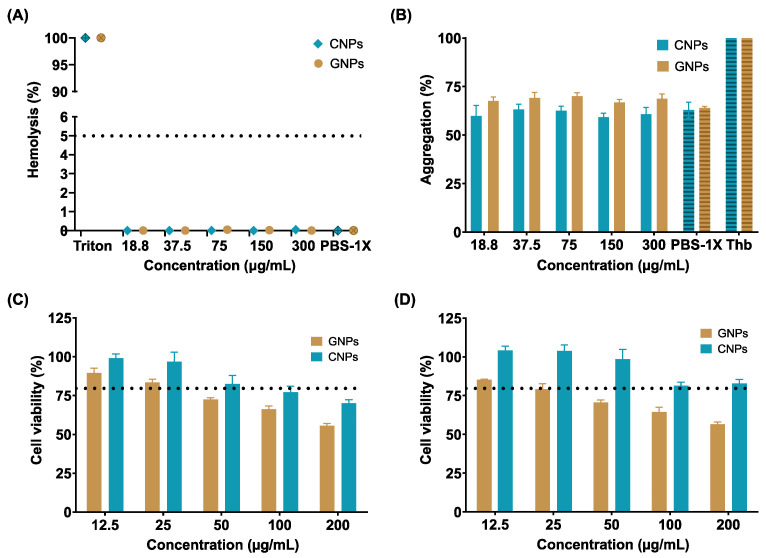
In vitro biocompatibility assays. (**A**) Hemolysis assay of GNPs and CNPs. Non-hemolytic behavior was found. (**B**) Platelet aggregation assay of GNPs and CNPs. In all cases, aggregation tendency was close to the negative reference (PBS-1X). MTT Cytotoxicity assay in Vero cells of GNPs and CNPs after 24 h (**C**) and 48 h (**D**). The reduction in cell viability (below 80%) is most likely due to massive internalization.

Cytotoxicity was assessed by the MTT assay after 24 (Figure 4C) and 48 h (Figure 4D). The results indicate a decrease in cell viability starting at 25 μg/mL with an average reduction of about 10% between treatments. The reduced cell viability could be attributed to the high affinity of the NPs toward cell membranes and their polysaccharide nature, which facilitates their rapid and massive internalization, most likely leading to compromised membranes and triggering apoptotic pathways [12]. In particular, innate and accelerated internalization has been reported for polysaccharide-based structures given their chemical composition, resembling common proteins, peptides, and metabolites [69]. These results suggest that a significant decrease in the administered dose will be needed to achieve the same efficacy in cargo transport and delivery, which confirms the potential of the obtained NPs as potent carriers and cell-penetrating vehicles.

Potential hemocompatibility was evaluated to estimate the impact of the NPs as blood-contacting nanodevices since intravenous (IV) administration remains their primary administration route to achieve a faster adsorption of therapeutic agents [70].

Figure 4A shows that even for the maximum tested concentration (200 µg/mL) of NPs, the hemolysis percentage remained below 1%, complying with international standard ISO10993-4. In addition, the platelet aggregation (Figure 4B) was found close to the negative control (PBS 1X), indicating that the IV administration of the NPs is likely to not trigger major thrombus formation processes [70].

The hemocompatibility of the NPs is explained by their resemblance to the extracellular matrix (ECM) polysaccharides, proteins, and peptides [71]. Additionally, some authors have reported that natural-polymer based NPs exhibit superior performance as blood-contacting materials because their superficial charges and degradation byproducts maintain the ionic balance of blood, present low-affinity with platelets receptors (thereby avoiding binding and activation), and showed no significant interference with red blood cell function [72,73]. The obtained hemocompatibility results were also close to those previously reported for LMW chitosan and type B gelatin [73,74,75].

## 4. Conclusions

Type B gelatin nanoparticles (GNPs) and LMW chitosan nanoparticles (CNPs) were successfully synthesized and purified with an alternative downstream processing method, achieving synthesis yields of 92.60% ± 0.66% and 89.94% ± 1.13% for CNPs and GNPs, respectively. To our knowledge, these results are on average 8% greater than those reported previously as the maximum and represent a cost-effective way to further study their scalability to produce nanostructured materials for the encapsulation and immobilization of therapeutic agents for delivery through different administration routes.

TEM results showed that GNPs and CNPs exhibited a nominal size of about 5 nm and 2 nm, respectively. However, in aqueous suspension, they tend to form aggregates of about 244 nm and 373 nm, similar to the behavior exhibited by many carriers suspended in solution such as liposomes where the van der Waals interactions play a major role [76,77]. Additionally, it has been reported that the formation of NP aggregates is related to the interactions between themselves, thereby causing polydispersity and precipitation [78,79]. The zeta-potential results for CNPs (+36.60 ± 3.25 mV) and GNPs (−13.42 ± 1.16 mV) confirm sufficient colloidal stability and the potential for electrostatic interactions with cell membranes, which are beneficial for their internalization and delivery of bioactive molecules. Further uptake assays should be conducted to verify this potential in vitro and elucidate the corresponding mechanistic details and efficiencies.

Biocompatibility assessment confirms the relatively low cytotoxicity of NPs since cell viability remained at about 50% (GNPs) and 75% (CNPs) after 24 and 48 h for the maximum tested concentration (200 µg/mL). This could be most likely attributed to their high affinity for cells membranes, causing massive internalization and consequently the triggering of apoptotic routes. This strongly suggests that if used as cell-penetrating agents, only low doses of the NPs might be needed to achieve a sufficiently high therapeutic effect and effective delivery. Additionally, the hemolysis percentage was below 1%, and platelet aggregation was close to the negative reference (PBS 1X), confirming their potential for a safe intravenous administration.

Overall, the newly developed PNP synthesis scheme not only provides high yields, but leads to nanoplatforms with considerable potential for drug and bioactive molecule delivery applications, as evidenced by their attractive physicochemical properties and the mild biological responses induced.

## References

[1] Ahmad M.B., Lim J.J., Shameli K., Ibrahim N.A., Tay M.Y. (2011). Synthesis of Silver Nanoparticles in Chitosan, Gelatin and Chitosan/Gelatin Bionanocomposites by a Chemical Reducing Agent and Their Characterization. Molecules.

[2] Tolaymat T.M., El Badawy A.M., Genaidy A., Scheckel K.G., Luxton T.P., Suidan M. (2010). An evidence-based environmental perspective of manufactured silver nanoparticle in syntheses and applications: A systematic review and critical appraisal of peer-reviewed scientific papers. Sci. Total Environ..

[3] Bharathala S., Sharma P., Maurya P.K., Singh S. (2019). Biomedical Applications of Nanoparticles. Nanotechnology in Modern Animal Biotechnology: Concepts and Applications.

[4] Spicer C.D., Jumeaux C., Gupta B., Stevens M.M. (2018). Peptide and protein nanoparticle conjugates: Versatile platforms for biomedical applications. Chem. Soc. Rev..

[5] Liu X., Wu R., Li Y., Wang L., Zhou R., Li L., Xiang Y., Wu J., Xing L., Huang Y. (2021). Angiopep-2-functionalized nanoparticles enhance transport of protein drugs across intestinal epithelia by self-regulation of targeted receptors. Biomater. Sci..

[6] Foroozandeh P., Aziz A.A. (2018). Insight into Cellular Uptake and Intracellular Trafficking of Nanoparticles. Nanoscale Res. Lett..

[7] Nugent S., Kumar D., Rampton D., Evans D. (2001). Intestinal luminal pH in inflammatory bowel disease: Possible determinants and implications for therapy with aminosalicylates and other drugs. Gut.

[8] Termsarasab U., Cho H.J., Kim D.H., Chong S., Chung S.J., Shim C.K., Moon H.T., Kim D.D. (2013). Chitosan oligosaccharide-arachidic acid-based nanoparticles for anti-cancer drug delivery. Int. J. Pharm..

[9] Bolhassani A., Javanzad S., Saleh T., Hashemi M., Aghasadeghi M.R., Sadat S.M. (2014). Polymeric nanoparticles. Human Vaccines Immunother..

[10] Elsabahy M., Wooley K.L. (2012). Design of polymeric nanoparticles for biomedical delivery applications. Chem. Soc. Rev..

[11] Hörter D., Dressman J.B. (2001). Influence of physicochemical properties on dissolution of drugs in the gastrointestinal tract. Adv. Drug Deliv. Rev..

[12] Shishir M.R.I., Xie L., Sun C., Zheng X., Chen W. (2018). Advances in micro and nano-encapsulation of bioactive compounds using biopolymer and lipid-based transporters. Trends Food Sci. Technol..

[13] Ensign L.M., Cone R., Hanes J. (2012). Oral drug delivery with polymeric nanoparticles: The gastrointestinal mucus barriers. Adv. Drug Deliv. Rev..

[14] Tsai L.C., Chen C.H., Lin C.W., Ho Y.C., Mi F.L. (2019). Development of mutlifunctional nanoparticles self-assembled from trimethyl chitosan and fucoidan for enhanced oral delivery of insulin. Int. J. Biol. Macromol..

[15] Rana V., Sharma R., Hussain C.M. (2019). Recent Advances in Development of Nano Drug Delivery. Applications of Targeted Nano Drugs and Delivery Systems.

[16] Hathout R.M., Metwally A.A. (2019). Gelatin Nanoparticles. Methods Mol. Biol..

[17] Zarif M.-E. (2018). A review of chitosan-, alginate-, and gelatin-based biocomposites for bone tissue engineering. Biomater. Tissue Eng. Bull..

[18] Sudarshan N.R., Hoover D.G., Knorr D. (2009). Antibacterial action of chitosan. Food Biotechnol..

[19] Ong S.Y., Wu J., Moochhala S.M., Tan M.H., Lu J. (2008). Development of a chitosan-based wound dressing with improved hemostatic and antimicrobial properties. Biomaterials.

[20] Aranaz I., Mengibar M., Harris R., Panos I., Miralles B., Acosta N., Galed G., Heras A. (2009). Functional Characterization of Chitin and Chitosan. Curr. Chem. Biol..

[21] Dhawan S., Singla A.K., Sinha V.R. (2004). Evaluation of mucoadhesive properties of chitosan microspheres prepared by different methods. AAPS PharmSciTech.

[22] Yang J., Tian F., Wang Z., Wang Q., Zeng Y.-J., Chen S.-Q. (2008). Effect of chitosan molecular weight and deacetylation degree on hemostasis. J. Biomed. Mater. Res..

[23] Rao J.P., Geckeler K.E. (2011). Polymer nanoparticles: Preparation techniques and size-control parameters. Prog. Polym. Sci..

[24] Zielińska A., Carreiró F., Oliveira A.M., Neves A., Pires B., Venkatesh D.N., Durazzo A., Lucarini M., Eder P., Silva A.M. (2020). Polymeric Nanoparticles: Production, Characterization, Toxicology and Ecotoxicology. Molecules.

[25] Croisier F., Jérôme C. (2013). Chitosan-based biomaterials for tissue engineering. Eur. Polym. J..

[26] Menon M.M. (2019). Moldable Hydrogel Formed from Oppositely Charged Gelatin Nanoparticles.

[27] Elzoghby A.O., Elgohary M.M., Kamel N.M. (2015). Implications of Protein- and Peptide-Based Nanoparticles as Potential Vehicles for Anticancer Drugs. Adv. Protein Chem. Struct. Biol..

[28] Vinjamuri B.P., Papachrisanthou K., Haware R.V., Chougule M.B. (2021). Gelatin solution pH and incubation time influences the size of the nanoparticles engineered by desolvation. J. Drug Deliv. Sci. Technol..

[29] Ahsan S.M., Rao C.M. (2017). The role of surface charge in the desolvation process of gelatin: Implications in nanoparticle synthesis and modulation of drug release. Int. J. Nanomed..

[30] Castro A. (2014). Formulación, Síntesis, Optimización y Caracterización de dos Tipos de Nanosistemas de Encapsulamiento Basados en Quitosano.

[31] Food and Drug Administration Regulations.gov. https://www.regulations.gov/document/FDA-2013-D-0350-0002.

[32] Khan S.A., Schneider M. Nanoprecipitation versus two step desolvation technique for the preparation of gelatin nanoparticles. Proceedings of the Colloidal Nanocrystals for Biomedical Applications VIII.

[33] Khan S.A. (2020). Opportunities and challenges in the techniques used for preparation of gelatin nanoparticles. Pak. J. Pharm. Sci..

[34] Chin S.F., Azman A., Pang S.C. (2014). Size controlled synthesis of starch nanoparticles by a microemulsion method. J. Nanomater..

[35] Salatin S., Barar J., Barzegar-Jalali M., Adibkia K., Kiafar F., Jelvehgari M. (2017). Development of a nanoprecipitation method for the entrapment of a very water soluble drug into Eudragit RL nanoparticles. Res. Pharm. Sci..

[36] Kowalczyk B., Lagzi I., Grzybowski B.A. (2011). Nanoseparations: Strategies for size and/or shape-selective purification of nanoparticles. Curr. Opin. Colloid Interface Sci..

[37] Geh K.J., Hubert M., Winter G. (2016). Optimisation of one-step desolvation and scale-up of gelatine nanoparticle production. J. Microencapsul..

[38] Shamarekh K.S., Gad H.A., Soliman M.E., Sammour O.A. (2019). Towards the production of monodisperse gelatin nanoparticles by modified one step desolvation technique. J. Pharm. Investig..

[39] Manoj N., Dinesh M., Vaibhav D., Narendra Kumar J. (2008). Development, characterization, and toxicity evaluation of amphotericin B-loaded gelatin nanoparticles. Nanomedicine.

[40] Carvalho J.A., Abreu A.S., Ferreira V.T.P., Gonçalves E.P., Tedesco A.C., Pinto J.G., Ferreira-Strixino J., Junior M.B., Simioni A.R. (2018). Preparation of gelatin nanoparticles by two step desolvation method for application in photodynamic therapy. J. Biomater. Sci..

[41] Balthasar S., Michaelis K., Dinauer N., Von Briesen H., Kreuter J., Langer K. (2005). Preparation and characterisation of antibody modified gelatin nanoparticles as drug carrier system for uptake in lymphocytes. Biomaterials.

[42] Khramtsov P., Burdina O., Lazarev S., Novokshonova A., Bochkova M., Timganova V., Kiselkov D., Zamorina S., Rayev M. (2021). Modified desolvation method enables simple one-step synthesis of gelatin nanoparticles from different gelatin types with any bloom values. Pharmaceutics.

[43] Khan S.A., Ali H., Ihsan A., Sabir N. (2015). Tuning the size of gelatin nanoparticles produced by nanoprecipitation. Colloid J..

[44] Leo E., Vandelli M.A., Cameroni R., Forni F. (1997). Doxorubicin-loaded gelatin nanoparticles stabilized by glutaraldehyde: Involvement of the drug in the cross-linking process. Int. J. Pharm..

[45] Lee E.J., Khan S.A., Park J.K., Lim K.-H. (2011). Studies on the characteristics of drug-loaded gelatin nanoparticles prepared by nanoprecipitation. Bioprocess. Biosyst. Eng..

[46] Das R.P., Chakravarti S., Patel S.S., Lakhamje P., Gurjar M., Gota V., Singh B.G., Kunwar A. (2020). Tuning the pharmacokinetics and efficacy of irinotecan (IRI) loaded gelatin nanoparticles through folate conjugation. Int. J. Pharm..

[47] Lu Z., Yeh T.-K., Tsai M., Au J.L.-S., Wientjes M.G. (2004). Paclitaxel-Loaded Gelatin Nanoparticles for Intravesical Bladder Cancer Therapy. Clin. Cancer Res..

[48] Lazaridou M., Christodoulou E., Nerantzaki M., Kostoglou M., Lambropoulou D.A., Katsarou A., Pantopoulos K., Bikiaris D.N. (2020). Formulation and In-Vitro Characterization of Chitosan-Nanoparticles Loaded with the Iron Chelator Deferoxamine Mesylate (DFO). Pharmaceutics.

[49] Thakur A. Taranjit Preparation of chitosan nanoparticles: A study of influencing factors. Proceedings of the AIP Conference.

[50] Fàbregas A., Miñarro M., García-Montoya E., Pérez-Lozano P., Carrillo C., Sarrate R., Sánchez N., Ticó J.R., Suñé-Negre J.M. (2013). Impact of physical parameters on particle size and reaction yield when using the ionic gelation method to obtain cationic polymeric chitosan–tripolyphosphate nanoparticles. Int. J. Pharm..

[51] Mahmood M.A., Madni A., Rehman M., Rahim M.A., Jabar A. (2019). Ionically Cross-Linked Chitosan Nanoparticles for Sustained Delivery of Docetaxel: Fabrication, Post-Formulation and Acute Oral Toxicity Evaluation. Int. J. Nanomed..

[52] Zhang K., Xu Y., Lu L., Shi C., Huang Y., Mao Z., Duan C., Ren X., Guo Y., Huang C. (2021). Hydrodynamic cavitation: A feasible approach to intensify the emulsion cross-linking process for chitosan nanoparticle synthesis. Ultrason. Sonochem..

[53] Patil P., Bhoskar M. (2014). Optimization and Evaluation of Spray Dried Chitosan Nanoparticles Containing Doxorubicin. Int. J. Curr. Pharm. Res..

[54] Demirbolatİsmail G.M., Degim İ.T. (2013). Preparation of chitosan nanoparticles by nano spray drying technology. J. Pharm. Sci..

[55] Ngan L.T.K., Wang S.-L., Hiep Đ.M., Luong P.M., Vui N.T., Đinh T.M., Dzung N.A. (2014). Preparation of chitosan nanoparticles by spray drying, and their antibacterial activity. Res. Chem. Intermed..

[56] Bernstein-Levi O., Ochbaum G., Bitton R. (2016). The effect of covalently linked RGD peptide on the conformation of polysaccharides in aqueous solutions. Colloids Surf. B. Biointerfaces.

[57] Tsereteli L., Grafmüller A. (2017). An accurate coarse-grained model for chitosan polysaccharides in aqueous solution. PLoS ONE.

[58] Van Vlierberghe S., Graulus G.J., Samal S.K., Van Nieuwenhove I., Dubruel P., Netti P.A. (2014). Porous hydrogel biomedical foam scaffolds for tissue repair. Biomedical Foams for Tissue Engineering Applications.

[59] Aramwit P., Jaichawa N., Ratanavaraporn J., Srichana T. (2015). A comparative study of type A and type B gelatin nanoparticles as the controlled release carriers for different model compounds. Mater. Express.

[60] Honary S., Zahir F. (2013). Effect of Zeta Potential on the Properties of Nano-Drug Delivery Systems-A Review (Part 1). Trop. J. Pharm. Res..

[61] Date A.A., Hanes J., Ensign L.M. (2016). Nanoparticles for oral delivery: Design, evaluation and state-of-the-art. J. Control. Release Off. J. Control. Release Soc..

[62] Sundar S., Kundu J., Kundu S.C. (2010). Biopolymeric nanoparticles. Sci. Technol. Adv. Mater..

[63] Moraru C., Mincea M., Menghiu G., Ostafe V. (2020). Understanding the Factors Influencing Chitosan-Based Nanoparticles-Protein Corona Interaction and Drug Delivery Applications. Molecules.

[64] Debnath S.K., Saisivam S., Debanth M., Omri A. (2018). Development and evaluation of Chitosan nanoparticles based dry powder inhalation formulations of Prothionamide. PLoS ONE.

[65] Ilaiyaraja N., Aishwarya D., Farhath K. (2015). Chlorogenic acid loaded chitosan nanoparticles with sustained release property, retained antioxidant activity and enhanced bioavailability. Asian J. Pharm. Sci..

[66] Seo Y.C., Choi W.Y., Lee C.G., Cha S.W., Kim Y.O., Kim J.C., Drummen G.P.C., Lee H.Y. (2011). Enhanced immunomodulatory activity of gelatin-encapsulated Rubus coreanus Miquel nanoparticles. Int. J. Mol. Sci..

[67] Ing L.Y., Zin N.M., Sarwar A., Katas H. (2012). Antifungal activity of chitosan nanoparticles and correlation with their physical properties. Int. J. Biomater..

[68] Azarmi S., Huang Y., Chen H., McQuarrie S., Abrams D., Roa W., Finlay W.H., Miller G.G., Löbenberg R. (2006). Optimization of a two-step desolvation method for preparing gelatin nanoparticles and cell uptake studies in 143B osteosarcoma cancer cells. J. Pharm. Pharm. Sci. Publ. Can. Soc. Pharm. Sci. Soc. Can. Sci. Pharm..

[69] De Anda-Flores Y., Carvajal-Millan E., Campa-Mada A., Lizardi-Mendoza J., Rascon-Chu A., Tanori-Cordova J., Martínez-López A.L. (2021). Polysaccharide-Based Nanoparticles for Colon-Targeted Drug Delivery Systems. Polysaccharides.

[70] Bianchera A., Bettini R. (2020). Polysaccharide nanoparticles for oral controlled drug delivery: The role of drug–polymer and interpolymer interactions. Expert Opin. Drug Deliv..

[71] Pan D.C., Myerson J.W., Brenner J.S., Patel P.N., Anselmo A.C., Mitragotri S., Muzykantov V. (2018). Nanoparticle Properties Modulate Their Attachment and Effect on Carrier Red Blood Cells. Sci. Rep..

[72] Pan D., Vargas-Morales O., Zern B., Anselmo A.C., Gupta V., Zakrewsky M., Mitragotri S., Muzykantov V. (2016). The Effect of Polymeric Nanoparticles on Biocompatibility of Carrier Red Blood Cells. PLoS ONE.

[73] Jesus S., Marques A.P., Duarte A., Soares E., Costa J.P., Colaço M., Schmutz M., Som C., Borchard G., Wick P. (2020). Chitosan Nanoparticles: Shedding Light on Immunotoxicity and Hemocompatibility. Front. Bioeng. Biotechnol..

[74] Heise K., Hobisch M., Sacarescu L., Maver U., Hobisch J., Reichelt T., Sega M., Fischer S., Spirk S. (2018). Low-molecular-weight sulfonated chitosan as template for anticoagulant nanoparticles. Int. J. Nanomed..

[75] Bello A.B., Kim D., Kim D., Park H., Lee S.-H. (2020). Engineering and Functionalization of Gelatin Biomaterials: From Cell Culture to Medical Applications. Tissue Eng. Part B Rev..

[76] Singh N., Kushwaha P., Ahmad U., Abdullah M., Singh N., Kushwaha P., Ahmad U., Abdullah M. (2020). Proliposomas: Una aproximación para el desarrollo de liposoma estables. ARS Pharm..

[77] Çağdaş M., Sezer A.D., Bucak S., Sezer A.D. (2014). Liposomes as Potential Drug Carrier Systems for Drug Delivery. Application of Nanotechnology in Drug Delivery.

[78] Witharana S., Hodges C., Xu D., Lai X., Ding Y. (2012). Aggregation and settling in aqueous polydisperse alumina nanoparticle suspensions. J. Nanopart. Res..

[79] Musino D., Genix A.-C., Chaussée T., Guy L., Meissner N., Kozak R., Bizien T., Oberdisse J. (2018). Aggregate Formation of Surface-Modified Nanoparticles in Solvents and Polymer Nanocomposites. Langmuir.

